# In Troubled Waters: Applying DNA Barcoding to Monitor Singapore's Shark Fin Trade

**DOI:** 10.1002/ece3.71607

**Published:** 2025-06-22

**Authors:** Anya Ramanan, Kimberly H. Quek, Nicole Chung Mae Sze, Nicole Isabel Oo Xinyen, David Kim Hyun Soo, Changjun Sung, Vivien Dimitrov, Rebekah P. Nix, Ming Han Mark Sng, Pei Xuan Jody Lim, Elisa X. Y. Lim, Benjamin J. Wainwright

**Affiliations:** ^1^ Yale‐NUS College National University of Singapore Singapore Singapore; ^2^ Department of Biological Sciences National University of Singapore Singapore Singapore

**Keywords:** CITES, DNA barcoding, IUCN, shark fin, sharks, wildlife trade

## Abstract

The global fin trade poses a significant threat to shark populations; many species of shark are at risk of extinction due to overfishing and unsustainable practices. This study examines the fin trade in Singapore, a globally significant fin trading hub, market and transit point. Using DNA barcoding techniques, we attempted to determine the species of origin for 300 processed fins that could not be identified by visual techniques. Fins were collected from a variety of outlets across Singapore. We identified 12 species, eight of which were identified as threatened on the IUCN Red List of threatened species (critically threatened *n* = 2, endangered *n* = 4 & vulnerable *n* = 2). Of all the samples we identified to the species or genus level, 13 (12 species and 1 entire genus) are listed on CITES Appendix II. This listing means that international trade has to be controlled to prevent further population declines and utilisation incompatible with their survival. Ninety‐eight percent of all the identifications made in this work belonged to species that are listed on CITES Appendix II. Demonstrating the importance of regular and repeated monitoring, we identified the blackchin guitarfish (*Glaucostegus cemiculus*); this is the first occurrence of fins from this species within Singapore and the wider Southeast Asian region. This is a CITES Appendix II listed species and one that has been designated as critically endangered by the IUCN. Without repeated monitoring, the presence of this species in Singpaore would likely have gone undetected.

## Graphical abstract

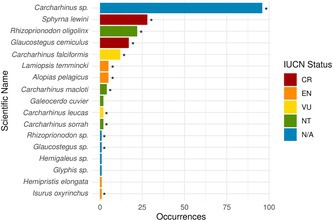

## 1 Introduction

More than a third of all chondrichthyans, which includes sharks, rays and chimaeras, are estimated to be at risk of extinction (Dulvy et al. 2021). Although broadly threatened by overfishing, bycatch and habitat loss, the shark fin trade remains a significant factor that contributes to these declines (Dulvy et al. 2021; Cardeñosa et al. 2022). The fin trade is a lucrative business, with the total declared value of world fin exports in 2011 being US$ 438.6 million (Dent and Clarke 2015). It is estimated that 26–73 million sharks are finned and traded annually to support this industry (Dulvy et al. 2014), with Asia recognised as a major consumer and market of fins. Throughout Asia, the primary demand for shark fin comes from shark fin soup, where this dish is considered a status symbol and sign of wealth (Dulvy et al. 2014; Drescher et al. 2022; Choy and Wainwright 2022).

The continuing global decline of shark populations is increasingly a conservation concern, sharks perform important ecological functions and help in the maintenance of ecosystem stability, and act as nutrient vectors that can influence primary production (Sherman, Simpfendorfer, et al. 2023). For example, grey reef sharks ( *Carcharhinus amblyrhynchos* ) have a diet largely consisting of pelagic fish, through consumption of prey and subsequent defecation, nutrients such as nitrogen and phosphorous are transferred to reef environments where they stimulate primary production (Sherman, Simpfendorfer, et al. 2023).

In efforts to prevent unsustainable exploitation, the trade of sharks is regulated through the Convention on International Trade in Endangered Species of Wild Fauna and Flora (CITES), whereas their conservation status is continually assessed by the International Union for Conservation of Nature (IUCN). CITES has three appendices designed to exert varying levels of trade control. The Appendix III lists species that are protected in at least one country, which has requested CITES' help in regulating trade (CITES n.d.). The CITES Appendix II lists species that are not necessarily threatened with extinction, but trade is controlled to prevent utilisation incompatible with their existence (CITES n.d.), whereas Appendix I includes species that are threatened with extinction and international trade of these species is only allowed in exceptional circumstances (CITES n.d.). The IUCN Red List is a checklist of taxa that have undergone extinction risk assessments (IUCN SSC Shark Specialist Group, n.d.). These species are then placed into a number of categories based upon their assessed risk (e.g., least concern, near threatened, vulnerable, endangered and critically endangered and so forth) (IUCN SSC Shark Specialist Group, n.d.).

Singapore is a significant consumer, and a central hub in the global trade of shark fins and the larger wildlife trade, with numerous examples of CITES listed species sold throughout the country (Dent and Clarke 2015; Drescher et al. 2022; Saigal et al. 2024; Liu et al. 2021; Boon 2017). The country ranks second in terms of shark fin imports and exports by value, trailing only behind Hong Kong (Dent and Clarke 2015; Liu et al. 2021; Boon 2017). Between 2000 and 2011, Singapore accounted for approximately 10% of global shark fin imports and 9% of exports, handling over 2000 t per year (Drescher et al. 2022; Boon 2017). Processed shark fins entering Singapore originate from countries across the globe, including but not limited to, Indonesia, India, Argentina and Spain. They are then re‐exported to major consumer markets, particularly those in China and Hong Kong, where the continuing demand for shark fin remains strong (Liu et al. 2021; Teo 2015). In Asia, shark fin consumption is rooted in Chinese cultural and social customs (Saigal et al. 2024; Choy et al. 2024). Shark fin soup, a traditional dish frequently served at weddings and other significant gatherings, is regarded as a symbol of wealth and social standing (Saigal et al. 2024; Choy et al. 2024; Dell'Apa et al. 2014; Zhou et al. 2021). The dish's popularity is driven not only by its cultural and symbolic value but also by belief in its alleged health benefits, consequently these norms continue to sustain consumer interest (Saigal et al. 2024; Choy et al. 2024; Dell'Apa et al. 2014; Zhou et al. 2021; Fabinyi 2012; Clarke et al. 2007). Despite growing awareness and the removal of shark fin soup from the menus of many dining establishments, a consequence of public pressure, its consumption in Singapore remains substantial (Choy et al. 2024; Murillo Rengifo et al. 2024; Yeo 2022). For example, despite increasingly negative perceptions surrounding the consumption of shark fin soup, it is still widely available in restaurants, and dried fins are easily purchased in markets, traditional Chinese medicinal shops and dried goods retailers throughout the country (Saigal et al. 2024).

A major obstacle that prevents effective regulation of the shark fin trade is the difficulty associated with correctly identifying what species a fin came from, which becomes more challenging once the fins have been processed for sale (Liu et al. 2021; Bornatowski et al. 2014). The processing and drying of fins remove essential morphological features, often making it impossible to accurately determine the species of origin through visual means (Drescher et al. 2022; Van Houtan et al. 2020). These difficulties compromise the ability of regulatory bodies to determine the species involved in the trade, heightening the risk that endangered or regulated species will unknowingly be introduced into supply chains (Drescher et al. 2022). This undermines attempts to enforce sustainable fishing practices (Liu et al. 2021) and protect at‐risk populations and species. Additionally, different species of shark are known to bioaccumulate toxic metals such as mercury, lead, cadmium and arsenic at varying rates (Chan et al. 2023). Consequently, not knowing what species a fin came from could expose consumers to harmful levels of toxic metals and create significant public health risks (Saigal et al. 2024; Chan et al. 2023; Garcia Barcia et al. 2020). To overcome the difficulties associated with species identification, molecular techniques have been developed, applied and proven to be reliable identification methods (Garcia Barcia et al. 2020; Shen et al. 2024). Here we used DNA barcoding and publicly accessible DNA databases to accurately determine the species that a processed fin came from (But et al. 2020; Nejati et al. 2024; Gaye et al. 2024).

Repeated surveys such as this, that build upon previous work (Saigal et al. 2024; Liu et al. 2021; Segura et al. 2023) are important; they provide a baseline through which any changes in species composition can be assessed (Wainwright et al. 2018; Muhala et al. 2024; Zangaro et al. 2024; Sheth and Thaker 2017). They also allow the impact and effectiveness of regulations to be determined as new policy designed to protect and conserve threatened species such as sharks, rays and guitar fish is introduced.

## 2 Methods

A total of 300 shark fins were purchased from Traditional Chinese Medicine shops (TCM) and dried goods stores throughout Singapore in August 2024. A list of all shops selling shark fins was compiled, and 30 shops were randomly selected to purchase fins. From each shop, a minimum of 10 fins were haphazardly chosen for purchase from a variety of size categories. DNA extraction was performed with a Qiagen DNeasy Blood and Tissue kit. Other than a modified final elution step that used 50 μL of elution buffer, all steps followed the manufacturer's protocol. To minimise potential contamination issues, each fin was carefully cleared of any loose tissue, and all apparatus used to handle and cut fins were wiped with ethanol between samples.

We targeted an approximate 350 bp fragment of the mitochondrial COI (cytochrome c oxidase subunit I) gene using the forward primer mlCOIintF (5′‐GGW ACW GGW TGA ACW GTW TAY CCY CC‐3′) (Leray et al. 2013) and reverse primer LoboR1 (5′‐TAA ACY TCW GGR TGW CCR AARAAY CA‐3′) (Lobo et al. 2013). Each PCR was performed in a 25 μL volume containing 12.5 μL GoTaq mastermix green, 1 μL forward primer (10 μM), 1 μL reverse primer (10 μM), 1 μL of Bovine Serum Albumin (BSA) (20 mg/mL), 7.5 μL nuclease‐free water and 2 μL of undiluted DNA template. The thermal cycling profile consisted of: 5 repeats of 94°C for 30 s, 48°C for 2 min, 72°C for 1 min, then 35 repeats of 94°C for 30 s, 54°C for 2 min, 72°C for 1 min, then a final extension step at 72°C for 5 min (Wainwright et al. 2018). A 1% TAE agarose gel was used to confirm successful DNA amplification prior to sequencing. PCR products were enzymatically cleaned and Sanger sequenced by Bio‐Basic Asia Pacific Pte Inc.

Sequences were visualised with Geneious v2024.0.7 (Kearse et al. 2012) and identified using the Barcode of Life Data System v4 (BOLD, https://www.boldsystems.org), and the Nucleotide BLAST (BLAST) function in Genbank (http://www.ncbi.nlm.nih.gov). We considered identifications positive if BOLD indicated a solid match with no known closely allied congeneric species currently identified, and the same species ID was made in GenBank with a 100% match. For sequences that could not be identified to the species level, the top matching genus in both databases was recorded. Only sequences that had clear well‐defined peaks and contained no ambiguous base calls were used in the identification step. CITES and IUCN designations were determined using the following online resources; CITES (https://checklist.cites.org/#/en) and IUCN (https://www.iucnredlist.org/).

## 3 Results

**FIGURE 1:**
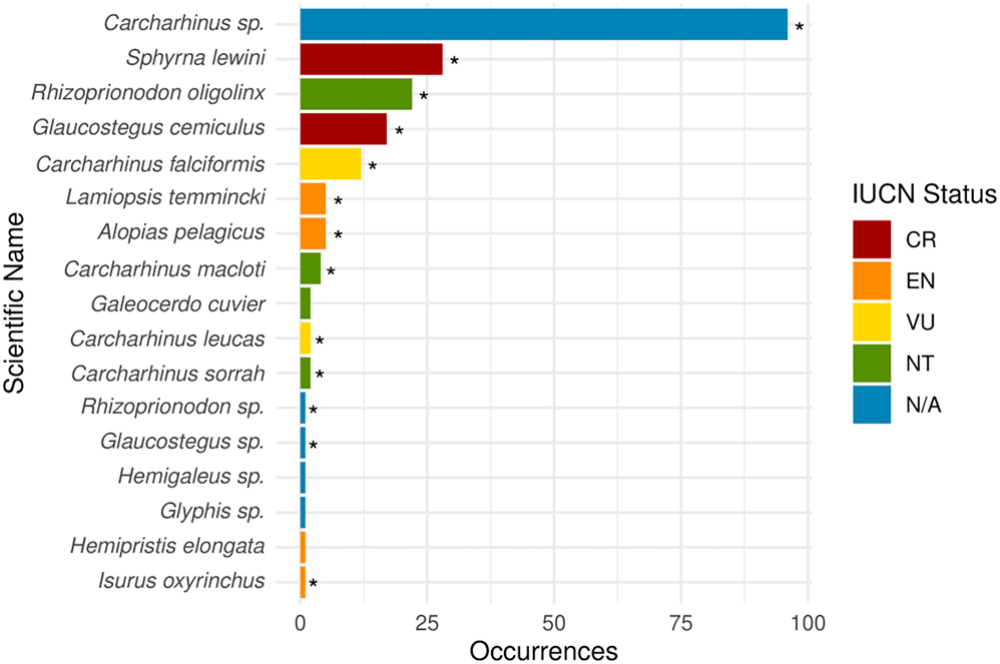
Bar chart detailing species or genus identification, occurrence and IUCN status. An asterisk (*) indicates CITES Appendix II listed species. An asterisk next to a genus level identification indicates that the species identified within this genus are listed on Appendix II.

We successfully barcoded 201 out of 300 samples at either the genus or species level; 99 fins could not be barcoded due to amplification failure or low sequence quality. All sequences used in identification were between 94 and 343 bp in length and had mean and median lengths of 277 and 283 bp, respectively. In total, we were able to identify 11 species of shark and one species of guitar fish. It was not possible to identify 100 fins beyond the level of genus. Using the criteria defined in the methods section, 96 fins could not be resolved beyond the *Carcharhinus* genus; identifications in this genus could belong to the following species: *Carcharhinus brevipinna* , *Carcharhinus falciformis* , *Carcharhinus limbatus* , *Carcharhinus tilstoni* , *Carcharhinus amblyrhynchoides* and *Carcharhinus leiodon*. Members of the identified *Glyphis* genus potentially belong to *Glyphis gangeticus* , *Glyphis fowlerae* or *Glyphis siamensis*. Samples identified as belonging to the *Hemigaleus* genus matched *Hemigaleus australiensis* and *Hemigaleus microstoma* , whereas *Glaucostegus* sp. identifications are either *Glaucostegus thouin* or *Glaucostegus typus*, with members of the genus *Rhizoprionodon identified as either Rhizoprionodon terraenovae* or *Rhizoprionodon porosus* . Of all identifications made at the species and genus levels, 13 are listed as CITES Appendix II species (Table 1 and Figure 1). The IUCN Red List describes two species as critically endangered, four as endangered, two as vulnerable and four as near threatened.

**TABLE 1:** Species identification, common names, occurrence, IUCN Red List status and CITES status.

Scientific name	Common name	Occurrence	CITES status	IUCN status
*Carcharhinus* sp.	Requiem Sharks	96	Appendix II	N/A
*Sphyrna lewini*	Scalloped Hammerhead	28	Appendix II	CR
*Rhizoprionodon oligolinx*	Grey Sharpnose Shark	22	Appendix II	NT
*Glaucostegus cemiculus*	Blackchin Guitarfish	17	Appendix II	CR
*Carcharhinus falciformis*	Silky Shark	12	Appendix II	VU
*Alopias pelagicus*	Pelagic Thresher	5	Appendix II	EN
*Lamiopsis temmincki*	Broadfin Shark	5	Appendix II	EN
*Carcharhinus macloti*	Hardnose Shark	4	Appendix II	NT
*Carcharhinus sorrah*	Spot‐tail Shark	2	Appendix II	NT
*Carcharhinus leucas*	Bull Shark	2	Appendix II	VU
*Galeocerdo cuvier*	Tiger Shark	2	N/A	NT
*Isurus oxyrinchus*	Shortfin Mako Shark	1	Appendix II	EN
*Hemipristis elongata*	Snaggletooth Shark	1	N/A	EN
*Glyphis* sp.	River Sharks	1	N/A	N/A
*Hemigaleus* sp.	Weasel Sharks	1	N/A	N/A
*Glaucostegus* sp.	Giant Guitarfishes	1	Appendix II	N/A
*Rhizoprionodon* sp.	Sharpnose Sharks	1	Appendix II	N/A

Abbreviations: CR, critically endangered; EN, endangered; LC, least concern; NT, near threatened; VU, vulnerable.

## 4 Discussion

The shark fin trade in Singapore and the broader Asian region continues to be driven by a high demand for shark fins, particularly in TCM practices (de Mitcheson et al. 2018). Singapore is a key transit hub in the global shark fin trade, with large volumes of fins passing through the country on their way to consumers in Hong Kong and mainland China (Boon 2017; Rodenbiker et al. 2023). Despite regulations designed to control this trade and prevent unsustainable exploitation, the enforcement of these rules is complicated by the many challenges enforcement officers and customs officials face when trying to correctly identify the species a processed fin came from. Further complicating this, many fins are sold under generic and all‐encompassing names such as ‘dried seafood’, ‘dried product’ or ‘dried goods’ (de Mitcheson et al. 2018). Despite an increasing global awareness surrounding the unsustainability of the fin trade, only 12% of shark fisheries are considered potentially sustainable (Rodenbiker et al. 2023). The vast majority of the fins entering the market come from unsustainable and often illegal fisheries, where overfishing and indiscriminate harvesting of vulnerable species contribute to the rapid decline of shark populations (Drescher et al. 2022). With 25,000 tons of dried fins traded each year, countries face a critical, but difficult challenge in balancing established cultural practices with urgent conservation needs (de Mitcheson et al. 2018).

Here, we successfully identified 201 samples to the species or genus level. We were unable to amplify DNA from a number of samples, or they produced low‐quality sequences that could not be used in identification. It is likely these samples contained DNA that was degraded through processing or through prolonged storage at ambient temperatures in shops. This is a commonly encountered problem when working with dried fins, more so when the hot and humid climate of Singapore is considered (Saigal et al. 2024; Shen et al. 2024).

The most commonly identified species in this work, *Sphyrna lewini* (Scalloped hammerhead), is listed on CITES Appendix II and identified as critically endangered on the IUCN Red list. From a conservation perspective, this fact is concerning. Hammerhead sharks have slow growth rates, a lengthy gestation period and are already identified as one of the most critically endangered shark species globally (Drescher et al. 2022). *S. lewini* is regularly identified in previous barcoding studies performed in Singapore (Drescher et al. 2022; Saigal et al. 2024; Liu et al. 2021; Shen et al. 2024). Compounding the negative effects that the fin trade has on *S. lewini* populations, it is frequently landed as bycatch, where, even if released it has a very high rate of mortality, with more than 90% not surviving when released (Consortium for Wildlife Bycatch Reduction, n.d.). On account of the high value that *S. lewini* fins can command (Mundy‐Taylor and Crook 2013; Abercrombie et al. 2005), it is estimated that 1.3–2.7 million individuals of this species are traded globally each year to supply the fin trade (Clarke et al. 2006).

We were unable to identify 96 fins beyond the *Carcharhinus* genus; this is a consequence of the limited resolving power of the COI gene, and this challenge is especially apparent when attempting to make identifications from short fragments of DNA. The degraded nature of DNA extracted from processed fins necessitates use of primers that target short fragments of DNA. These are well‐known challenges and have been encountered in multiple fin barcoding studies (Drescher et al. 2022; Saigal et al. 2024; Liu et al. 2021; Shen et al. 2024). Nevertheless, this is still important information; all Carcharhinidae are currently listed on CITES Appendix II, meaning a degree of control on their trade is exerted to prevent utilisation incompatible with their survival. Without DNA barcoding, even genus‐level identifications on these fins would be challenging. *C. falciformis* was the most commonly identified Carcharhinid in our study, better known as the Silky Shark. This species is highly migratory and found throughout tropical waters, ranging from the Western Atlantic to the Eastern Pacific and various parts of the Indo‐Pacific, including the Red Sea and Hawaiian Islands (FishBase, n.d.). *C. falciformis* is a viviparous and placental species that gives birth to 3–13 pups per litter (Hussey et al. 2010; Grant et al. 2018). This low fecundity makes it vulnerable to overfishing and other unsustainable practices where it is targeted for its fins, meat, skin to make leather products and liver oil. On account of this, it is one of the most commonly encountered species in the shark fin trade (FishBase, n.d.; Li et al. 2023), and over the past two decades, *C. falciformis* has been documented to be one of the most commonly traded species in the markets of Hong Kong and China (Cardeñosa et al. 2021).

Highlighting the value of repeated surveys such as that performed here, *Glaucostegus cemiculus* (Blackchin Guitarfish) was one of the most frequently identified species in this work. This species has not been identified in multiple surveys previously performed in Singapore (Drescher et al. 2022; Saigal et al. 2024; Liu et al. 2021; Shen et al. 2024; Wainwright et al. 2018), and it is probable that without this work, its presence within the Singapore trade would go unnoticed. The presence of *G. cemiculus* is noteworthy as it has a limited geographic range, found predominantly in coastal regions of the Atlanto‐Mediterranean region (Akyol and Capapé 2014). As such, its detection in the markets of Singapore demonstrates the complexity of the underlying global trade networks that supply retailers and ultimately consumers with shark fins. This species is specifically targeted by fishers across its range, a consequence of its large and highly prized fins (Notarbartolo di Sciara et al. 2016). This targeted fishing pressure has led to its listing on the IUCN Red List as Critically Endangered and its inclusion on the CITES Appendix II as a trade regulated species. Additionally, *G. cemiculus* tends to have nurseries that are in close proximity to shallow coastal waters (Bengi̇l et al. 2020), making this species an easy target for fishers seeking to capitalise on its much sought after and valuable fins (Notarbartolo di Sciara et al. 2016). Compounding these risks, its low fecundity and prolonged maturation period severely limits its ability to recover from population declines (Notarbartolo di Sciara et al. 2016). This is especially problematic given its very low resilience and reported minimum population doubling time of more than 14 years (Notarbartolo di Sciara et al. 2016). Further repeated monitoring is required to determine whether this was a chance encounter, or its presence is more indicative of the sourcing of fins from further afield as other species become rarer and more challenging to find as they are fully exploited.

## 5 Conclusion

Similar to previous work performed in Southeast Asia, we show that endangered species continue to be sold within the markets of Singapore. The majority of the 201 samples we identified are listed on the IUCN Red List of threatened species as critically endangered, endangered or vulnerable. To mitigate the impact of this trade, effective monitoring, correct labelling and the application of advances in identification tools at key checkpoints would help to prevent the import and subsequent consumption of endangered species. Given Singapore's significance in, and the key role the country plays as a global transit hub in the shark fin trade, the country has the opportunity to play an oversized and important role in preventing the unsustainable exploitation of sharks on a global scale. Ultimately, continued and improved efforts to monitor and regulate the trade are crucial in preventing the extinction of endangered sharks and rays.

## Author Contributions

**Anya Ramanan:** data curation (equal), formal analysis (equal), investigation (equal), methodology (equal), writing – original draft (lead), writing – review and editing (equal). **Kimberly H. Quek:** conceptualization (equal), data curation (equal), investigation (equal), methodology (equal), writing – original draft (lead), writing – review and editing (equal). **Nicole Chung Mae Sze:** data curation (equal), formal analysis (equal), investigation (equal), methodology (equal), writing – original draft (lead), writing – review and editing (equal). **Nicole Isabel Oo Xinyen:** data curation (equal), formal analysis (equal), investigation (equal), methodology (equal), writing – original draft (lead), writing – review and editing (equal). **David Kim Hyun Soo:** data curation (equal), formal analysis (equal), investigation (equal), methodology (equal), writing – original draft (lead), writing – review and editing (equal). **Changjun Sung:** data curation (equal), formal analysis (equal), investigation (equal), methodology (equal), writing – original draft (lead), writing – review and editing (equal). **Vivien Dimitrov:** data curation (equal), formal analysis (equal), investigation (equal), methodology (equal), writing – original draft (lead), writing – review and editing (equal). **Rebekah P. Nix:** data curation (equal), formal analysis (equal), investigation (equal), methodology (equal), writing – original draft (lead), writing – review and editing (equal). **Ming Han Mark Sng:** data curation (equal), formal analysis (equal), investigation (equal), methodology (equal), writing – original draft (lead), writing – review and editing (equal). **Pei Xuan Jody Lim:** data curation (equal), formal analysis (equal), investigation (equal), methodology (equal), writing – original draft (lead), writing – review and editing (equal). **Elisa X. Y. Lim:** project administration (equal), supervision (equal), writing – review and editing (equal). **Benjamin J. Wainwright:** conceptualization (lead), data curation (equal), formal analysis (equal), investigation (equal), methodology (equal), project administration (equal), writing – original draft (supporting), writing – review and editing (lead).

## Conflicts of Interest

The authors declare no conflicts of interest.

## Supporting information


Table S1.

## Data Availability

All sequences used in species identification are available in Table S1.
